# Oral Administration of Resveratrol-Loaded Solid Lipid Nanoparticle Improves Insulin Resistance Through Targeting Expression of SNARE Proteins in Adipose and Muscle Tissue in Rats with Type 2 Diabetes

**DOI:** 10.1186/s11671-019-3042-7

**Published:** 2019-07-09

**Authors:** Roohollah Mohseni, Zahra ArabSadeghabadi, Nasrin Ziamajidi, Roghayeh Abbasalipourkabir, Azam RezaeiFarimani

**Affiliations:** 1 Department of Clinical Biochemistry, School of Medicine, Hamadan University of Medical Sciences, Hamadan, Iran; 2 Student Research Committee, Hamadan University of Medical Sciences, Hamadan, Iran; 3 Department of Basic Medical Sciences, Neyshabur University of Medical Sciences, Neyshabur, Iran

**Keywords:** Nanoparticles, Resveratrol, Insulin resistance, SNARE proteins

## Abstract

In the current study, we developed resveratrol (RES)-loaded solid lipid nanoparticle (SLN-RES) in order to improve insulin resistance through the upregulation of SNARE protein complex in rats with type 2 diabetes. The SLN-RES characteristics include the following: the average size of 248 nm, the zeta potential of − 16.5 mV, and 79.9% RES entrapment efficiency. The release profile of SLN-RES showed an initial burst followed by a sustained release in natural condition. Infrared spectroscopy results revealed good incorporation of RES into core SLN. Spherical nanoparticle with less aggregation was observed under electronic microscopic examination. Oral administration of SLN-RES prevented weight loss and showed better hypoglycemic effect than RES. Serum oxidative stress status was restored to the normal level by SLN-RES. Furthermore, expression of synaptosomal-associated protein 23 (Snap23), syntaxin-4 (Stx4), and vesicle-associated membrane protein 2 (Vamp2) as the major elements of SNARE protein complex were reduced by SLN-RES more significantly than RES treatment in muscle tissue. However, SLN-RES has a similar effect to RES treatment in adipose tissue. Taken together, our results revealed SLN-RES could be a modern and interestingly therapeutic approach for the improvement of insulin resistance through targeting the expression of Snap23, Stx4, and Vamp2 in adipose and muscle tissues.

## Presentation of the Hypothesis

The encapsulation of resveratrol (RES) into lipid core nanoparticles enhanced its benefits through improving stability and oral intestinal absorption when consumed orally. SLN-RES improves insulin resistance more than RES. Antioxidant effect of RES increases when incorporated into SLNs.

## Testing the Hypothesis

Spectroscopic analysis of SLN-RES was performed in order to determine encapsulation efficacy. The second hypothesis was to evaluate the effect of SLN-RES treatment on the oxidative stress parameters and its hypoglycemic effect against type 2 diabetes in animal models.

## Implications of the Hypothesis

Increased stability and intestinal absorption lead to the bioavailability of RES when administrated orally. Increased bioavailability of RES when incorporated into SLNs leads to increased concentration of RES in targeted tissues. Increased concentration of RES in targeted tissues leads to more hypoglycemic effect of RES when incorporated into SLNs than RES.

## Background

Type 2 diabetes mellitus is a metabolic disorder that is characterized by insulin resistance. Insulin resistance is mainly developed through the disruption of glucose transport system (GLUT 2 and 4) in muscle and adipose tissue [1]. The SNARE proteins immobilized GLUT 4 system to the cell membrane and play important roles in the membrane trafficking, docking, and vesicle fusion. The synaptosomal-associated protein 23 (SNAP-23), syntaxin-4 (STX4), and vesicle-associated membrane protein 2 (VAMP-2) are known as the main component of SNARE protein complex [2]. Previous reports identified that the downregulation of SNARE proteins accelerates insulin resistance [3]. Over the past years, a large body of data showed herbal components have many beneficial effects that can improve many metabolic disorders [4–6]. Research attempt by Côté et al. showed the acute intraduodenal infusion of RES compensated insulin resistance via decreasing duodenal SIRT1 protein and reducing hepatic glucose production in the rat model [4]. It is also, Gencoglu et al. reported the intraperitoneal administration of RES relieved insulin resistance in diabetic rats via normalizing the expression of visfatin and upregulating the SIRT1 expression in skeletal muscle. RES feeding leads to increase GLUT4 and GLUT2 expression in streptozotocin-induced diabetes in rats. Overall, these findings provided novel insights into the beneficial effects of RES supplementation against insulin resistance. Despite its promising therapeutic application, low intestinal absorption and gastrointestinal degradation mainly lead to low bioavailability of RES when administered orally [7].

Over the last years, nanomedicine provides an intelligent approach for drug delivery of many drugs in order to overcome its low bioavailability, poor stability, and enhancement of targeting strategies [8]. As such, micelles, liposomes, polymeric nanoparticles, and solid lipid nanoparticles (SLNs) are the most famous drug delivery systems [9, 10]. Recent studies showed incorporating RES into nanoscale delivery systems is a convenient method to circumvent its limitation and could be more effective than the pure drug suspension in clinical attempts [11]. Recent evidence showed incorporation of drugs in solid lipid nanoparticles (SLNs) could increase the bioavailability of RES when administrated orally [11, 12]. Frozza et al. showed nanoencapsulation of RES improved its neuroprotective effect against Alzheimer’s diseases via increasing drug concentration in the brain tissue [13]. According to the previous reports that were mentioned above, it has been proposed that the encapsulation of RES into lipid core nanoparticles can improve insulin resistance better than RES through the elevation of its oral bioavailability.

The present work focused towards the development, optimization, and characterization of RES-loaded SLN (SLN-RES) with a view to improve its oral bioavailability. Therefore, in the current study, we prepared SLN-RES and tried to determine its properties. Then, the effect of oral treatment of SLN-RES on the fasting blood sugar (FBS), insulin, and oxidative stress parameters was evaluated in rats with type 2 diabetes. Furthermore, we investigated the effect of oral administration of SLN-RES on the gene expression of Snap23, Stx4, and Vamp2 in adipose and muscle tissue.

## Materials and Methods

### Materials

Trans-resveratrol (> 99%) was provided from Mega Resveratrol (USA), streptozotocin (STZ) and nicotinamide (NA) were purchased from Sigma-Aldrich (Germany), and the hydrogenated soybean lecithin (S100) was given as a gift by Lipoid KG (Ludwigshafen, Germany). Hydrogenated palm oil (S154) was given as a gift by Condea (Witten, Germany), and TRIzol reagent and cDNA synthesis kit were supplied from Invitrogen (USA). Insulin Rat ELISA Kit was purchased from Bio-Equip (China). Other products and solvents were used in analytical grade.

### Preparation of SLN-RES

SLN-RES was produced according to the previous study with minor modifications [14]. Briefly, 40 mg S100 and 1 g sorbitol were added to a 15-ml distilled water and were heated at 70 °C as the aqueous phase. The organic phase including 100 mg S154, 70 mg S100, and RES was melted at 70 °C, and then, 5 ml chloroform was added as an organic solvent. Subsequently, the organic phase was quickly mixed with the preheated aqueous phase under stirring at 1000 rpm/min until the organic solvent was removed. The resultant suspension was sonicated for 2 min and then injected in cold water (2 °C) under continuous stirring at 1000 rpm/min for 2 h in order to fastly solidify the lipid matrix. Then, the sample was maintained at dark condition until it was dried. The resulting sample was washed twice with distilled water by centrifuging to remove the supernatant which contained the free drug. The supernatants were subjected to measurement of entrapment efficiency **(**EE). A series of SLN were prepared by adding a different amount of pure RES (30, 50, and 70 mg) into the molten lipid phase to achieve high EE. The effect of RES loading on the mean particle size, polydispersity index (PDI), surface charge (zeta potential), and EE of SLNs was evaluated (Table 1).

### Characterization of SLN-RES

The mean diameter nanoparticle, zeta potential, and PDI of SLN-RES were measured by laser diffraction (Zetasizer Nano-ZS; Malvern Instrument, UK). Characterization was performed after SLN-RES dilution in distilled water (1/30).

### Entrapment Efficiency

EE value is defined as the percentage of trapped RES into SLN relative to the total RES using the following equation. The amount of trapped RES was determined by separating free RES from encapsulated RES. Free RES was quantified using UV spectrophotometer at 310 nm.

$$ \mathrm{EE}\%\kern0.5em =\kern0.5em \left[\left(\mathrm{weight}\kern0.5em \mathrm{of}\kern0.5em \mathrm{total}\kern0.5em \mathrm{drug}-\mathrm{weight}\kern0.5em \mathrm{of}\kern0.5em \mathrm{untrapped}\kern0.5em \mathrm{drug}\right)/\left(\mathrm{weight}\kern0.5em \mathrm{of}\kern0.5em \mathrm{total}\kern0.5em \mathrm{drug}\right)\right]\times 100 $$

### In Vitro Drug Release Study

The in vitro drug release pattern of RES was analyzed as follows. The prepared SLN-RES was dissolved in plasma medium while stirring at pH 7.4 and 1.2. At the defined time points (0.5, 1, 2, 4, 6, and 8 h), the defined amount of medium was withdrawn and subsequently was filtered by a 0.24-μm syringe filter for the quantification of the free form of RES. The filtered RES represented the amount of RES that was released into the medium.

### Transmission Electron Microscopy

Morphological characterization was evaluated by transmission electron microscopy (TEM). Briefly, SLN-RES was spread onto a carbon-coated copper grid and viewed under TEM ZEISS. Surface morphology, aggregation, and irregularity of SLN-RES were characterized.

### Fourier Transform Infrared Spectroscopy

The S100, RES, and dried sample of SLN and SLN-RES were analyzed for confirming their intermolecular interactions and surface chemistry characterization of nanoparticles by Fourier transform infrared spectroscopy (FTIR), using an infrared spectrometer (FTIR PerkinElmer, USA). The spectra were obtained in the range of 400 and 4000 cm^−1^. The dried samples were prepared using KBr to form pellets.

### Animal Study Design

Twenty male Wistar rats (200–250 g) were provided from the Animal House of Razi Institute (Iran). The animals were handled according to the protocols approved by the Ethics Committee of Hamadan University of Medical Sciences. Rats were fed with fresh water and a standard chow and maintained under a controlled condition (25 ± 2 °C and lighting 12-h light/dark cycles). Type 2 diabetes was induced by intraperitoneal injection of STZ 65 mg/kg (0.1 M in sodium citrate; pH, 4.5) and nicotinamide 110 mg/kg at a single dose [15]. The FBS level was measured after 3 days. Rats with glucose levels above 150 were considered as diabetic models. After a week, RES and powdered SLN-RES were dissolved in distilled water and administrated orally by gavage daily for 1 month. The animals were randomly divided into four groups of 5 rats in each group: HC, healthy control; DC, diabetic control; RES, diabetic rat treated with 10 mg/kg of resveratrol orally; and SLN-RES, diabetic rat treated with resveratrol-loaded solid lipid nanoparticles orally (powdered SLN-RES sample containing 10 mg/kg RES). At the end of the study, the animals were weighed and then anesthetized with ketamine and xylazine intraperitoneally (100 mg/kg and 10 mg/kg, respectively). Afterward, blood was collected from cardiac puncture and serum separated and stored at − 20 °C. Subsequently, skeletal muscle and visceral adipose tissue were harvested and immediately frozen and kept at − 80 °C for further analysis.

### Measurement of Fasting Blood Sugar

The FBS was measured by a colorimetric assay kit (Pars Azmun, Iran).

### The Measurement of Serum Insulin

Serum level of insulin was measured by a commercial ELISA kit (Bio-Equip, China) according to the manufacturer’s instruction. Insulin resistance was calculated using the homeostasis model assessment (HOMA) formula.

### Total Antioxidant Capacity

The ability of the sample for reducing ferric (Fe^+3^) to ferrous (Fe^+2^) was determined as the total antioxidant capacity (TAC). The reaction between Fe^2+^ and 2,4,6-Tri(2-pyridyl)-s-triazine (TPTZ) leads to a blue-colored complex [16].

### Total Thiol Group

Total thiol groups (-SH) were measured using 5,5'-dithiobis(2-nitrobenzoic acid) (DTNB) reagent. This reagent reacts with thiol groups to produce a yellow-colored complex [17].

### Lipid Peroxidation Assay

Malondialdehyde (MDA) as the end product of lipid peroxidation process was measured by using the colorimetric method. The peroxidized lipids react with thiobarbituric acid (TBA) and produce a pink-colored complex. 1,1,3,3-Tetraethoxypropane was used as the standard [18].

### Total Oxidant Status

The oxidation potential of the sample was measured by the total oxidant status (TOS) method. Briefly, Fe^+3^ and xylenol orange produce a colored complex, in an acidic condition. The assay was calibrated with H_2_O_2_, and the results were expressed in μM H_2_O_2_ equivalent/L [19].

### Quantitative Reverse Transcription Polymerase Chain Reaction

Quantitative reverse transcription polymerase chain reaction (qRT-PCR) was performed in order to determine the gene expression of Snap23, Stx4, and Vamp2. Total mRNA was extracted from fast frozen adipose and muscle tissue using TRIzol reagent. The mRNA quantity and quality were determined using a NanoDrop UV spectrophotometer (BioTek Laboratories, Inc., USA). The mRNA was reverse transcribed to cDNA using the RevertAid First Strand cDNA Synthesis Kit. Quantitative amplification was performed with specific primer sequences using the CFX96 real-time PCR detection system. The forward and reverse primers respectively were used for gene amplification: 5′-dTTCCGTTTCTGTGTCCAATAG and 5′-dTTGTGCTTTCCAGAGACTCAT for Snap23, 5′-dTCAGCAGACTATGTGGAAC and 5′-dCCAAGATGAGAACAGTGACAGA for Stx4, and 5′-dCTACTTGGTCCTAAGAATCC and 5′-dCAGAAGAGTGAAGAGTAATGG for Vamp2. Relative change of gene expression was calculated according to 2^-ΔΔCt^ formula. 18s rRNA as housekeeping gene was used. All experiments were performed in triplicates.

### Statistical Analysis

Statistical analysis was performed by using SPSS 16 and GraphPad Prism 6.00 software, LaJolla, CA (USA). Data were expressed as mean ± standard deviation (SD). One-way analysis of variance (ANOVA) was used to compare differences between groups. *p* < 0.05 was considered as significant level.

## Results and Discussion

### Physicochemical Characterization of SLN-RES

As can be seen in Table 1, as the drug to lipid ratio increased, the particle size and PDI value were almost constant which could be due to the formation of multiple phospholipid layers in SLN [11]. The size range of present nanoparticle was about 250 nm which could be suitable for intestinal absorption and bypass liver and spleen filtration system [20]. Also, this size range can lead to the enhanced bioavailability of RES through increased circulation time of SLN-RES and prolonged drug release. The development of nanoparticles with smaller size and PDI value can facilitate their intestinal absorption. As a suggestion, the modification of SLN with polyethylene glycol (PEG) could improve our formulation to enhance their permeability and systemic circulation time [21].

As shown in Table 1, the PDI value of 0.4 is considered to have heterogeneous distribution, which expressed the presence of agglomerates. In SLN-RES-30, the elevation of RES content helps to increase the probability of being accommodated in the SLN, although the EE percentage was depleted obviously followed by increasing drug content in SLN-RES-70 formulation. It was concluded that increased lipid content in SLN-RES-70 is not sufficient to accommodate all amount of RES into SLN matrix [22]. So SLN-50 was selected as the optimized formulation for further investigations.

Zeta potential results showed a negative surface charge (− 16.5 ± 17.7 mV) for the current formulations. Regarding morphological observation, the surface charge of SLN-RES was considered sufficient for preventing particle aggregation phenomena and fine SLN stability [21]. The zeta potential of all formulations with or without drug was almost similar to each other meaning that the encapsulation of drug into SLN did not have a significant impact on the surface charge of the carrier. As a result, the drug was placed at the inside of the SLN due to the lipophilic nature of RES [23].

**Table 1:** The effect of the different ratio of drug to lipid on the SLN characteristic

Formulation name	RES (mg)	Average size (nm)	PDI	ZP (mV)	EE (%)
SLN	0	219.7	0.480	− 16.1	–
SLN-RES-30	30	257.6	0.447	− 17.7	64.3
SLN-RES-50	50	248.3	0.422	− 16.5	79.7
SLN-RES-70	70	224.3	0.474	− 17.3	60.1

*SLN-RES-30, 50, and 70* nanoparticle formulation containing 30, 50, and 70 mg of resveratrol, *RES* pure resveratrol powder, *PDI* polydispersity index, *ZP* zeta potential, *EE* entrapment efficiency

### The in Vitro Release Assay

**Fig. 1:**
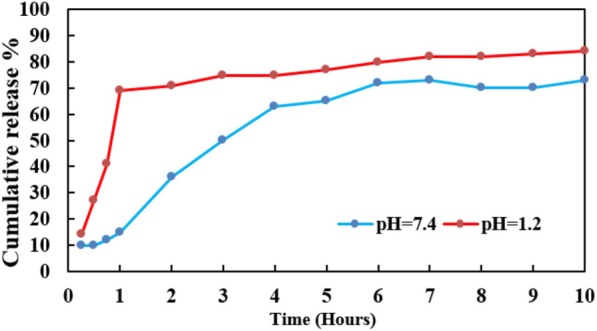
In vitro release profiles of RES from SLN-RES in acidic and natural conditions

As the results are presented in Fig. 1, different releasing behaviors were detected at pH = 7.4 and pH = 1.2. After 6 h and 1 h, about 70% of RES was released from nanoparticle at neutral and acidic pH conditions, respectively. The initial burst release was due to the release of drugs adsorbed on the surface of nanoparticles in the initial phase [24]. Afterward, a sustained release manner could be attributed to drug-lipid interaction, concentration gradient, and incorporation of drug into the oily carrier core. Sustained release profile leads to the enhanced drug serum concentration and consequently helps to increase the bioavailability and directed delivery of RES. As a suggestion, the administration of RES intravenously can bypass the acidic condition of the stomach [22].

### Morphology Evaluation

**Fig. 2:**
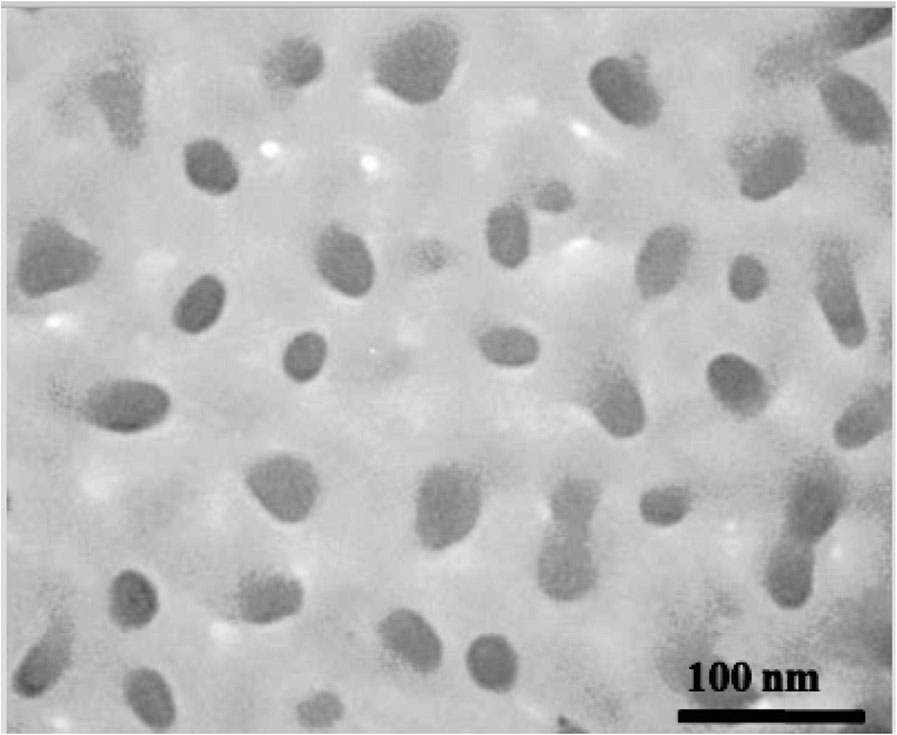
Transmission electron micrograph of SLN-RES. Bar=100 nm

As can be seen in Fig. 2, the TEM analysis gave rise to good results where most of the particles attained regular spherical shape and size in the range of 20–30 nm. Also, the rod-shaped and amorphous nanoparticles were observed accompanied with less aggregation. The particle size of SLN was estimated between 20 and 30 nm which disagree with the DLS findings (Table 1). This discrepancy may be a result of the hydration of nanoparticle through sample dilution in DLS analysis. Also, there was a rod-shaped nanoparticle which may be due to the constant injection of an emulsion into the cold water by a syringe needle. We did not observe SLN aggregations in TEM observation which represents enough surface charge and stability for our formulation [23].

### Validating the Loading Efficiency of RES by Infrared Spectroscopy

**Fig. 3:**
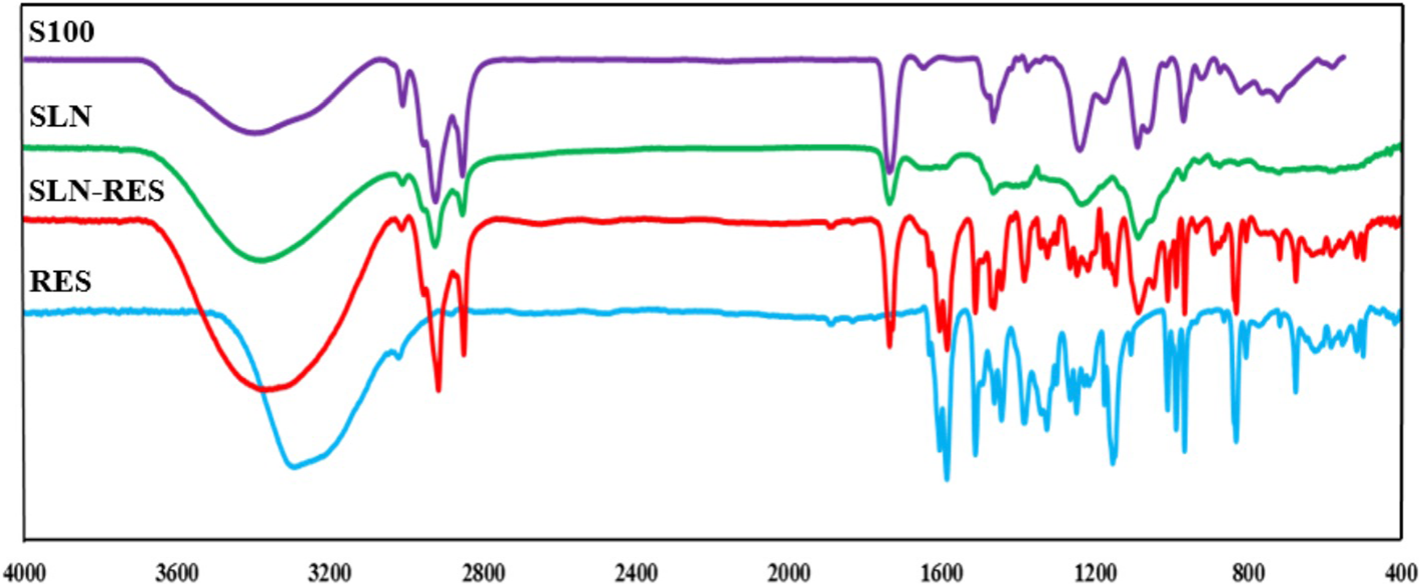
FTIR spectrum of SLN (solid lipid nanoparticle), SLN-RES (resveratrol-loaded solid lipid nanoparticle), S100 (lecithin), and RES (pure resveratrol powder)

As shown in Fig. 3, the FTIR results from lecithin spectrum revealed a broad peak at 3370–3390 cm^−1^ that refers to N-H symmetric stretch from choline functional group. A peak at 1735 cm^−1^ represented C=O stretching from ester compound in lecithin. Also, the characteristic peaks at 2922, 2853, and 3010 cm^−1^ are due to the stretching of C–H groups. The SLN spectrum exhibited several characteristic peaks similar to the lecithin spectrum with no shift, but the intensity of peaks belonging to lecithin was greatly decreased when placed into SLN. This may be due to the hydrophilic environment and the shielding effect of the surfactant. In the present study, RES spectrum exhibited functional group including absorption bands at 3290 cm^−1^ due to O-H stretching belonging to alcoholic group, 965 cm^−1^ for trans C=C bond, 1154 cm^−1^ for C-O stretching, 1445 cm^−1^ and 1587 cm^−1^ for C=C stretching in aromatic (AR) ring, and 1606 cm^−1^ for C-C stretching of alkene group. Also, the RES exhibited characteristic peaks with strong intensity of monosubstituted C-H bend at 770 cm^−1^ and Ar-C-H stretching at 3020 cm^−1^. There were peaks with intensity at 1175–1263 that represented the ArO-H group of RES. Our results validated the incorporation of RES into SLN whereas the RES fingerprint was repeated in SLN-RES, but the SLN spectrum was not associated with this fingerprint. Also, a peak around 1735–1740 cm^−1^ was attributed to C=O stretching (R-C(O)-O-R) that refers to ester in lecithin phospholipid in all spectrum expect to RES.

FTIR data validated the presence of drug in the carrier. There was a broad peak at 3040–3670 cm^−1^ in lecithin, SLN, and SLN-RES that was due to O-H stretching which contributes to the strong hydrophobic nature of samples. The broad peak shifting between SLN and SLN-RES may be referred to hydrogen bond formation when RES is placed into SLN. We concluded the particle surface was covered by lecithin because of the enhancement of C-H stretching in SLN-RES. On the other hand, the decreased intensity of RES fingerprint in SLN-RES spectrum refers to the lipid covering of RES in SLN structure. The difference between characteristic peaks from RES and SLN-RES validated that there is a potential chemical interaction between RES and other formulation components. A new peak that became apparent at 1000 cm^−1^ in SLN-RES was attributed to Ar-O-R that referred to the interaction between RES and lecithin. On the other hand, in RES spectrum, the absorption band at 1106 cm^−1^ was related to Ar-O-H group that disappeared in SLN-RES. This change provides another evidence for interacting between RES and SLN. Moreover, an increased intensity became apparent at 2850–2900 cm^−1^ in SLN-RES than in SLN indicating the enhancement of C-H stretching which may be due to increasing surface localization of surfactant and placing RES in the core nanoparticle. Overall, our results validated the incorporation of RES in lipid core SLN which is carved by lecithin [11, 22].

### The Effect of RES and SLN-RES Treatment on the Body Weight Gain, Fasting Blood Sugar, Insulin, and HOMA Index

The results in Table 2 showed that in the DC group, the STZ injection decreased the body weight significantly than in the HC group. RES administration prevented weight loss compared to the DC group. SLN-RES serves normal body weight more significantly than RES treatment whereas resorted to the HC group. Based on the previous reports, the oral administration of SLN-RES had a similar effect to free RES when administrated intraperitoneally. Gencoglu et al. reported that the intraperitoneal treatment of RES served the body weight after diabetes induction whereas the final body weight of diabetic models that received RES was 10% lower than that of the healthy rats [25].

**Table 2:** The effect of RES and SLN-RES treatment on the body weight gain, fasting blood sugar, insulin, and HOMA index in diabetic rat models

Group	DC	DC + RES	DC + SLN-RES
Body weight (g)	− 69.4 ± 14.4	− 49.9 ± 18.0^α+^	− 12.3 ± 2.8^α#π^
FBS (mg/dl)	+ 211.3 ± 92.1	+ 98.3 ± 38.6^α*^	+ 38.5 ± 13.6^α#δ^
Insulin (μU/ml)	− 4.0 ± 1.2	− 1.8 ± 0.9^α#π^	− 0.9 ± 0.2^α#π^
HOMA	+ 3.0 ± 1.1	+ 2.2 ± 0.8^α#^	+ 0.1 ± 0.04^α#πδ^

The alteration of parameters was expressed as compared with healthy control group. *DC* diabetic control, *RES* resveratrol treatment 10 mg/kg, *SLN-RES* resveratrol-loaded solid lipid nanoparticles containing 10 mg/kg of resveratrol, *FBS* fasting blood sugar, *HOMA* homeostasis model assessment-estimated insulin resistance. Data were expressed as mean ± SD

^α^Compared to the DC group

^δ^There was a significant difference between RES and SLN-RES group (*p* < 0.05)

^π^Restored to the HC group (there was no significant difference compared to the HC group (*p* > 0.05)).

*p < 0.05

^+^*p* < 0.01

^#^*p* < 0.001

As shown in Table 2, FBS was significantly elevated in the DC group compared with the HC group. At the end of the study, serum FBS was decreased by both RES and RES-SLN treatment compared to the DC group. Diabetes induction decreased the serum level of insulin compared to the HC group. Interestingly, the serum level of insulin in diabetic rats was compensated by RES-SLN treatment. The administration of RES and SLN-RES improved HOMA than DC group remarkably. In the SLN-RES group, HOMA was better than RES treatment which becomes near to the HC group.

The hypoglycemic effect obtained with the SLN-RES was better than RES significantly which may be due to the improved intestinal absorption and increased circulation time of nanoencapsulated RES in blood. Consistent with our proposal, Sadi et al. showed the intraperitoneal treatment of RES coincided with a profound hypoglycemic effect and an improved insulin resistance in STZ-induced diabetes [26].

### The Effect of RES and SLN-RES Treatment on the Serum Level of Oxidative Stress Parameters

As mentioned in Table 3, in the current study, diabetes induction leads to reduced antioxidant indicators and increased oxidant indicators significantly compared to the HC group. RES treatment prevented the depletion of serum TAC level and inhibited the elevation of MDA. Surprisingly, the SLN-RES treatment could restore serum TAC and MDA level completely. Probably, the increased circulation time of RES led to a better modulatory effect of RES-SLN [27]. In agreement with the present result, Gokce et al. reported that SLN and nanostructured lipid carriers (NLC) containing RES reduced intracellular ROS in cell culture fibroblast [28]. Also, Coradini and co-authors reported that the co-encapsulation of RES and curcumin in lipid carrier decreased the hydroxyl radical remarkably in vitro [29]. Moreover, the administration of RES-SLN led to decreased TOS value and increased -SH level as compared with the DC group significantly. However, RES treatment didn’t improve TOS and -SH level that showed the weak antioxidant effect of RES which might be attributed to the low bioavailability of RES when administered orally.

**Table 3:** The effect of RES and SLN-RES treatment on the oxidant and antioxidant indicators in diabetic rat models

Groups	HC	DC	RES	SLN-RES
TAC (mM)	0.26 ± 0.04	0.12 ± 0.01	0.18 ± 0.03^α*^	0.23 ± 0.01^α#πδ^
-SH (mM)	0.38 ± 0.06	0.17 ± 0.04	0.20 ± 0.06	0.25 ± 0.08^α+^
MDA (μM)	0.48 ± 0.12	1.24 ± 0.06	0.82 ± 0.12^α#^	0.59 ± 0.03^α#π^
TOS (μmol H_2_O_2_ equiv/l)	1.40 ± 0.13	1.95 ± 0.18	1.86 ± 0.15	1.59 ± 0.10^α#^

*HC* healthy control, *DC* diabetic control, *RES* resveratrol treatment 10 mg/kg, *SLN-RES* resveratrol-loaded solid lipid nanoparticles containing 10 mg/kg of resveratrol, *TAC* total antioxidant capacity, *-SH* total thiol group, *MDA* malondialdehyde, *TOS* total oxidant status. Data were expressed as mean ± SD

^α^Compared to the DC group

^δ^There was a significant difference between RES and SLN-RES group (*p* < 0.05)

^π^Restored to the HC group (there was no significant difference compared to the HC group (*p* > 0.05)).

*p < 0.05

+*p* < 0.01

*#p* < 0.001

### The Effect of RES and SLN-RES Treatment on the Gene Expression of Snap23, Stx4, and Vamp2 in Adipose Tissue

**Fig. 4:**
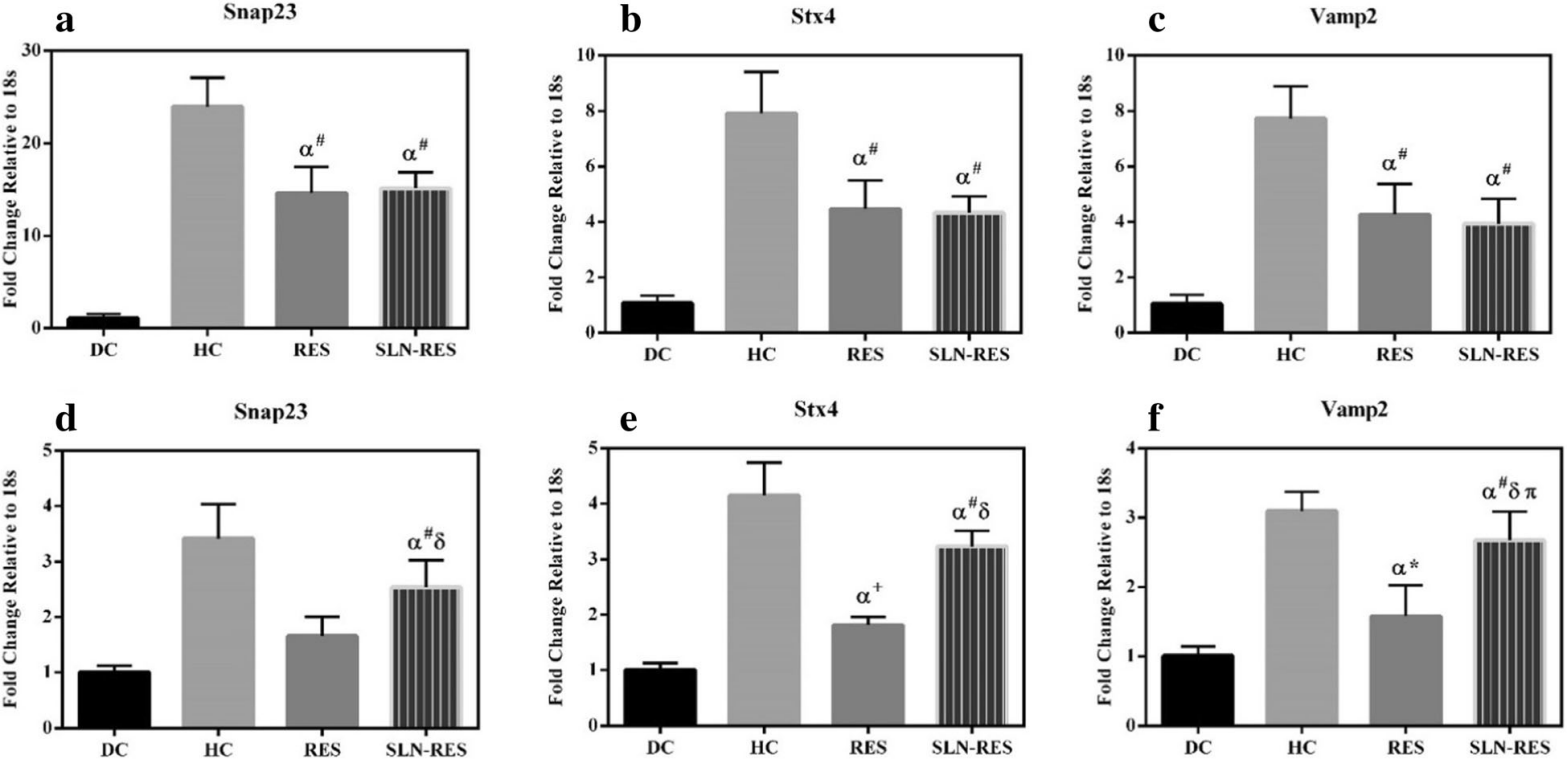
The effect of RES and SLN-RES treatment on the mRNA level of Snap23, Stx4, and Vamp2 in adipose (**a**–**c**) and muscle tissue (**d**–**f**). *α*, compared to the DC group; *δ*, there was a significant difference between RES and SLN-RES group (*p* < 0.05); *π*, restored to the HC group (there was no significant difference compared to the HC group (*p* > 0.05)). Data were expressed as mean ± SD.*p < 0.05, ^+^*p* < 0.01, ^#^*p* < 0.001

Previous attempts showed that the elevation of the gene expression of Snap23, Stx4, and Vamp2 led to improving insulin resistance. Oh et al. reported that the overexpression of Stx4 in the pancreatic cells of the transgenic model improved insulin resistance [30]. Here, the RES treatment induced the gene expression of Snap23, Stx4, and Vamp2 in adipose obviously (Fig. 4a–c). Farimani et al. showed that the RES treatment downregulated the gene expression of Stx4 and Vamp2 significantly in the adipose tissue of diabetic rat model [31]. Any significant changes were not observed between RES and SLN-RES treatment in adipose tissue in which probably the half-life of serum elimination of RES is not enough for adipose tissue drug exposure. The gene expression of Snap23 did not affect RES and SLN-RES which may be a result of the overproduction of SNAP23 protein in the adipocytes that can lead to transcriptional repression via a feedback mechanism. To address this question, the measurement of the protein level of SNAP23 could explain the present data in more details. Also, the measurement of hepatic Snap23 expression using time course method would explain our observation clearly.

### The Effect of RES and SLN-RES Treatment on the Gene Expression of Snap23, Stx4, and Vamp2 in Muscle Tissue

As illustrated in Fig. 4d–f, STZ injection in the DC group was associated with downregulation of Snap23, Stx4, and Vamp2 in muscle tissue compared to the HC group. SLN-RES induced the expression of Snap23 and Stx4 in muscle tissue better than the free form of RES. We observed the best result about the gene expression of Vamp2. The oral administration of SLN-RES compensated the downregulation of Vamp2 near to the normal level. Similar to our results, Mullainadhan et al. showed that the administration of bisphenol-A in adult male albino rats increased the protein expression of Snap23, Stx4, and Vamp2 in the gastrocnemius muscle [32]. Likely increased absorption of RES through RES-SLN administration may enhance the chance of RES-SLN to reach the muscle tissue that led to better regulatory effect on the gene expression of Snap23, Stx4, and Vamp2. Based on the previous evidence, the potential penetrating ability and cell uptake capacity of SLN-RES affect the tissue accumulation of RES that may be responsible for the different effects of SLN-RES on the gene expression of SNARE proteins in adipose and muscle tissue. In other words, our results supported differently in vivo biodistribution pattern of RES in intact form within the SLN.

## Conclusion

We prepared suitable nanocarrier in terms of physicochemical and morphological properties for oral delivery of RES. In light of our result, we concluded that SLNs could serve as a promising delivery system to enhance the therapeutic effect of oral treatment of RES against insulin resistance through improving the hypoglycemic effect and elevating the expression of Snap23, Stx4, and Vamp2 in adipose and muscle tissue. However, subsequent studies will be necessary to identify the in vivo biodistribution and pharmacokinetic properties of SLN-RES.

## Data Availability

All data generated or analyzed during this study are included in this published article.

## Abbreviations

RES: Resveratrol
SLN: Solid lipid nanoparticle
SLN-RES: Resveratrol-loaded solid lipid nanoparticle
SNAP-23: Synaptosomal-associated protein 23
STX4: Syntaxin-4
VAMP-2: Vesicle-associated membrane protein 2
TEM: Transmission electron microscopy
FTIR: Fourier-transform infrared spectroscopy
-SH: Total thiol group
TAC: Total antioxidant capacity
MDA: Malondialdehyde
TOS: Total oxidant status
